# Transcutaneous Vagus Nerve Stimulation Modulates EEG Microstates and Delta Activity in Healthy Subjects

**DOI:** 10.3390/brainsci10100668

**Published:** 2020-09-25

**Authors:** Lorenzo Ricci, Pierpaolo Croce, Jacopo Lanzone, Marilisa Boscarino, Filippo Zappasodi, Mario Tombini, Vincenzo Di Lazzaro, Giovanni Assenza

**Affiliations:** 1 Unit of Neurology, Neurophysiology, Neurobiology, Department of Medicine, University Campus Bio-Medico of Rome, via Álvaro del Portillo, 21, 00128 Rome, Italy; j.lanzone@unicampus.it (J.L.); m.boscarino@unicampus.it (M.B.); m.tombini@unicampus.it (M.T.); v.dilazzaro@unicampus.it (V.D.L.); g.assenza@unicampus.it (G.A.); 2 Department of Neuroscience, Imaging and Clinical Sciences, G. d’Annunzio University of Chieti-Pescara, 66100 Chieti, Italy; pierpaolo.croce@unich.it (P.C.); f.zappasodi@unich.it (F.Z.); 3 Institute for Advanced Biomedical Technologies (ITAB), G. d’Annunzio University of Chieti-Pescara, 66100 Chieti, Italy

**Keywords:** transcutaneous vagus nerve stimulation, microstates, EEG, power spectrum, delta activity

## Abstract

Transcutaneous vagus nerve stimulation (tVNS) is an alternative non-invasive method for the electrical stimulation of the vagus nerve with the goal of treating several neuropsychiatric disorders. The objective of this study is to assess the effects of tVNS on cerebral cortex activity in healthy volunteers using resting-state microstates and power spectrum electroencephalography (EEG) analysis. Eight male subjects aged 25–45 years were recruited in this randomized sham-controlled double-blind study with cross-over design. Real tVNS was administered at the left external acoustic meatus, while sham stimulation was performed at the left ear lobe, both of them for 60 min. The EEG recording lasted 5 min and was performed before and 60 min following the tVNS experimental session. We observed that real tVNS induced an increase in the metrics of microstate A mean duration (*p* = 0.039) and an increase in EEG power spectrum activity in the delta frequency band (*p* < 0.01). This study confirms that tVNS is an effective way to stimulate the vagus nerve, and the mechanisms of action of this activation can be successfully studied using scalp EEG quantitative metrics. Future studies are warranted to explore the clinical implications of these findings and to focus the research of the prognostic biomarkers of tVNS therapy for neuropsychiatric diseases.

## 1. Introduction

Direct electrical stimulation of the left cervical vagus nerve (VNS) is an invasive method for effectively treating otherwise medication-resistant epilepsy and depression [1,2,3], and its application is under investigation for a broad range of neuropsychiatric disorders [4]. Besides the recognized clinical efficacy of invasive VNS, there is evidence supporting additional potential benefits, such as the enhancement of cognition in Alzheimer’s disease, improvement of sleep patterns [5], and proposed antinociceptive and immunomodulatory effects [6]. However, the invasiveness required for its use has hindered the widespread diffusion of this technique. Indeed, VNS requires the surgical implantation of an electrical stimulator connected to an electrode located along the cervical branch of the vagus nerve. In recent years, to decrease the disadvantages of traditional VNS and its possible side-effects, an alternative approach for the non-invasive stimulation of the vagus nerve has been introduced [7]. Such a method involves the transcutaneous stimulation (tVNS) of the external auditory channel at the inner side of the tragus, in order to activate the auricular branch of the vagus nerve and, subsequently, the nuclei of the X cranial nerve sited in the brainstem. In detail, the nucleus of the solitary tract is a pivotal structure as it represents the target of major afferent vagal inputs and because it presents widespread projections toward a variety of key cerebral areas, including amygdala, thalamus, hippocampus and neocortex [8]. Still, the mechanism of action and potential effectiveness of tVNS with respect to diagnostics and the therapy of neurological and psychiatric disorders remains to be verified, and the functional neurobiology of how VNS, invasive or non-invasive, works is poorly understood [9,10,11]. Therefore, there is a need to further explore the potential influence of tVNS on cortical functional dynamics.

The electroencephalogram (EEG) is a sensitive and reliable tool for substantiating cortical functions and activities. In particular, each EEG frequency band power (i.e., delta, theta, alpha, beta, gamma) has a functional role [12]. Moreover, numerous previous works showed that the resting-state EEG can be represented as a sequence of “microstates”, which consist of discrete cortical topographies that remain constant for about 40–120 ms [13]. These EEG microstates derive from the synchronous activities of various cortical networks reflecting different functions [14]. As such, microstate analysis represents a promising approach to providing a global topographical representation of specific neural processes without any type of a priori hypothesis [15], as opposed to most of the EEG frequency analysis techniques, which aim at assessing the brain’s electrical field at a specific location or in specific frequency bands.

Along this line, the objective of this exploratory study was to investigate the possible effects of tVNS on cerebral cortex activity in healthy volunteers using resting-state microstates and power spectrum EEG analysis. We hypothesize that tVNS-induced modification in microstates and power spectrum may provide new insights into the neurophysiological mechanisms of action of tVNS, which will eventually contribute to the identification of potential neurophysiological biomarkers of VNS activity and efficacy.

## 2. Materials and Methods

### 2.1. Subjects

Eight male subjects aged 25–45 years (mean age: 30.5 ± 6.02 years) were recruited to the study. Female participants were excluded due to evidence of menstrual cycle-related effects on cortical excitability, which could introduce a possible confounding effect [16]. All the subjects were naïve to tVNS and had no previous knowledge of the neurophysiological non-invasive stimulation protocols of the vagus nerve. All subjects were right-handed according to the Edinburgh Handedness Inventory (laterality score ≥ 75%) [17]. Exclusion criteria included the use of central nervous system (CNS) drugs, a history of substances/drugs abuse (nicotine/alcohol included) and a history of psychiatric or neurologic diseases. Subjects were instructed to abstain from caffeine and alcohol starting from the day before the experimental session. Subjects provided informed consent, and the study was approved by the local ethical committee. The scientific protocol was approved by the Ethical Committee of our Institution at University Campus Bio-Medico, Via Alvaro del Portillo 200, 00128 Rome, Italy. Project Identification code: “tVNS_ROB_CS”, Version n. 1.0, Accepted on 07/23/2019. Registration code: 29/19 PAR ComEt CBM.

### 2.2. Experimental Design

This is a randomized sham-controlled double-blind study with a cross-over design. We performed real tVNS at the left external acoustic meatus, while sham stimulation was conducted over the left ear lobe. All subjects underwent both real and sham tVNS for 60 min. We pseudo-randomized the order by which the conditions were administered across healthy subjects. 48 h were reserved as the interval time between sessions. Participants were blind to the stimulation condition. The EEG features were tested before (Pre) and immediately after (Post) the exposure to tVNS or to sham stimulation, which both lasted 60 min.

### 2.3. tVNS Procedure

The bipolar stimulation of the auricular branch of the vagus nerve was carried out using an electric stimulator (Twister-EBM) and two Ag-AgCl electrodes (5 mm in diameter), placed at the left external acoustic meatus on the inner side of the tragus. The distance between the cathode and anode was 5 mm. For sham stimulation, electrodes were attached at the left ear lobe. We chose this anatomical area by following the previously described methodology of our previous work [18], since it is acknowledged to be outside the innervation of the auricular branch of the vagus nerve [19]. To diminish the risk of possible cardiac side-effects, we placed the electrodes on the left ear, in view of the fact that peripheral nerves directed to the heart are supposed to originate from the right vagus nerve [20].

tVNS was carried out as trains lasting 30 s and was composed of 600 pulses (intra-train pulse frequency = 30 Hz; pulse duration = 0.5 ms) repeated every 5 min for 60 min. We chose these parameters according to previous studies in animals [21] and humans [18]. The amplitude of stimulation was personally adjusted to a level reaching above the detection threshold and below pain perception. Across these amplitude levels, we used whenever possible an intensity of 8 mA as suggested by Polak et al. [22], who showed that such intensity can activate the vagus brainstem nuclei without perception of pain.

### 2.4. Safety Evaluation

Despite the fact that tVNS was performed on the left ear, dangerous cardiovascular side-effects can still be potentially experienced by subjects. Therefore, during the stimulation, subjects were strictly monitored for variations in the heart rate (HR) and blood pressure (BP). Such parameters were recorded every 15 min. Moreover, to verify the tolerability of tVNS, we asked the subjects every 5 min if they felt unpleasant sensations or discomfort.

### 2.5. EEG Recordings

The EEG activity was acquired from 32 scalp sites using a BrainAmp 32MRplus (BrainProducts GmbH, Munich, Germany), with electrodes positioned according to the 10-10 International System. Additional electrodes were used as ground and as reference. The ground electrode was positioned at Oz. The linked mastoid served as the reference for all the electrodes. To ensure wakefulness throughout the recording sessions, subjects were required to keep their eyes open and to fixate upon a target over the opposite wall. The signals recorded were bandpass filtered at 0.1–1000 Hz and digitized at a sampling rate of 5 kHz. Skin/electrode impedance was kept below 5 kOhms. Horizontal and vertical eye movements were detected by recording the electro-oculogram (EOG). The voltage between the reference electrodes and electrodes located beneath the right eye recorded vertical eye movements and blinks. The EEG recording lasted 5 min and was performed before and 60 min following the tVNS experimental session.

### 2.6. Data Analysis and Statistics

A computer scientist engineer (P.C.) and neuroscientists (L.R., G.A.) with expertise in EEG analysis and biostatistics performed the data processing and statistical analysis. The data processing followed two steps: (i) signal preprocessing and feature extraction, and (ii) statistical analysis. Signal preprocessing was conducted by using MATLAB 2019a version (MathWorks, Natick, MA, USA) and the EEGLAB signal processing library [23], whereas statistical analysis was conducted using the statistical software package R [24].

#### 2.6.1. EEG Data Analysis

For each subject, the raw 5 min EEG signal was visually inspected to remove very noisy channels or recording segments highly contaminated with movement artefacts. Data were then filtered between 1 and 70 Hz and notch filtered at 50 Hz (Butterworth filter of 2nd order, forward and back filtering). A semiautomatic procedure, based on Independent Component Analysis (ICA), was applied to remove ocular and cardiac artefacts [25]. Two types of analysis were then performed on EEG artefact-free resting state data: (i) Microstate Analysis and (ii) Power Spectral Density (PSD) estimation.

#### 2.6.2. Microstates Analysis

**Figure 1:**
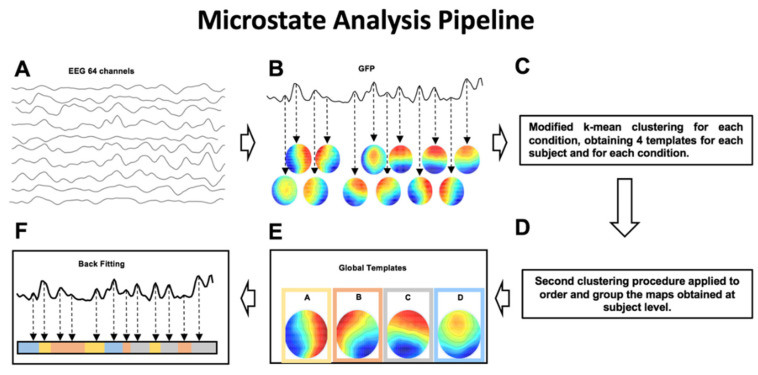
(**A**,**B**) Step 1: The intervals of stable topographical configurations are identified. (**C**–**E**) Step 2: The global templates of the dominant microstates are calculated for the identified intervals of brain functional stability. (**F**) Step 3: The identified global templates are backfitted to each noise-free EEG dataset to find the specific sequence of microstates on which metrics are calculated.

Microstates analysis aims at identifying the dominant topographical configurations (global templates or microstates) that alternate during the EEG time course to depict the ongoing brain dynamics [13]. Through quantitative metrics, it is then possible to calculate features characterizing the specific sequence of microstates, such as the mean duration, coverage and occurrence of each microstate. The sequence of steps necessary to perform microstate analysis is illustrated in Figure 1.

For each subject and for each condition, intervals of stable topographical configuration were identified. Specifically, we calculated, for each time instant of the EEG time course, the Global Field Power (GFP). GFP is defined as the standard deviation of the EEG signal amplitude across all electrodes at a given time instant and is a reference-independent descriptor of the potential field strength. GFP peaks can be considered as corresponding to intervals of highest topographic stability, when the probability of observing a transition to a different (stable) topographical configuration is lower [15]. For this reason, for each subject and for each condition, the scalp potential corresponding to the maximum values of GFP were fed to a modified version of a k-means clustering algorithm [26]. To identify the optimal number of microstate templates, we applied the clustering k-algorithm, varying k from 2 to 12. The optimal number of k was identified by applying the Krzanowski–Lai (KL) criterion [15]. For each condition, the centroids of the k clusters were then considered as a set of microstate templates. From this procedure 4 microstate templates are obtained for each subject and for each condition. By applying the same clustering procedure to the individual microstates template in each group (EEG-Pre-Real, EEG-Post-Real, EEG-Pre-Sham, EEG-Post-Sham), 4 microstate templates for each condition were obtained.

**Figure 2:**
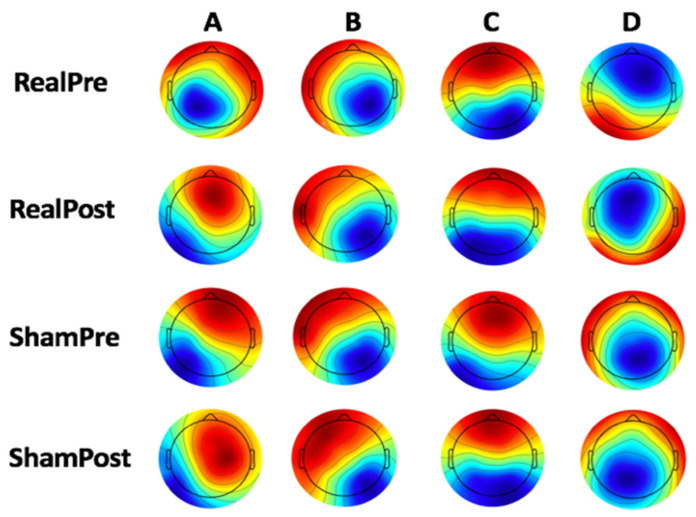
Microstates (**A**–**D**) template for each condition.

To identify the global microstate templates, for each group, the sets of individual microstate templates were averaged to calculate the microstate template for each condition (Figure 2).

**Figure 3:**
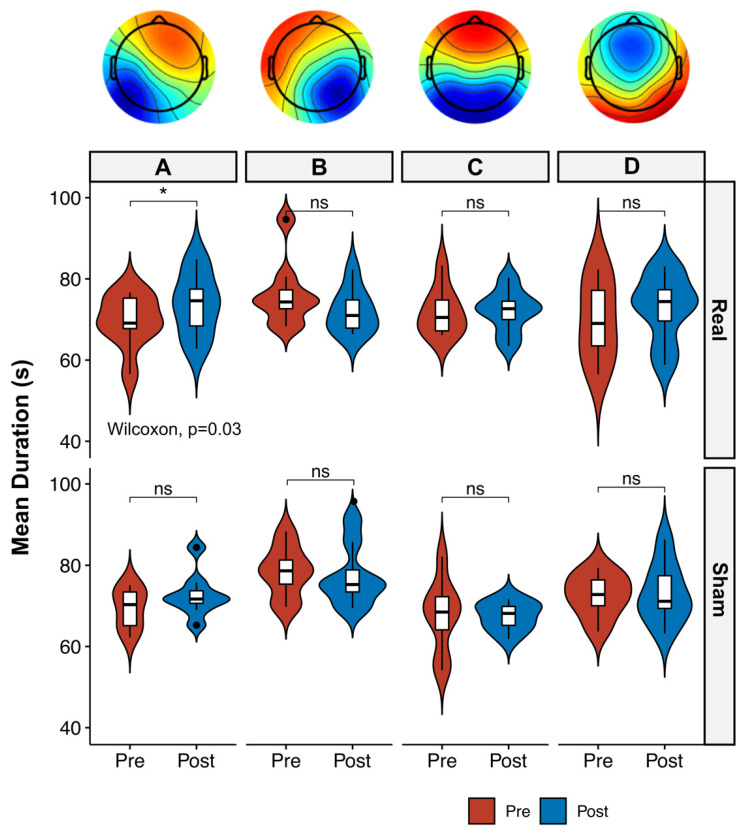
Boxplot and violin plot distributions of mean duration of the four microstate templates across time (Pre vs. Post) and conditions (Real vs. Sham). Circles denote values that are farther than 1.5 interquantile ranges. Microstate maps (from **A** to **D**, upper panel) represent the global microstate templates obtained from condition-wise microstate template. The A map was significantly longer in duration after Real tVNS stimulation, as opposed to Sham tVNS stimulation, which failed to reveal any significant difference. * = *p* < 0.05, *ns* = not significant.

Then, an iterative procedure was applied [27]: a number of microstate templates equal to k was randomly chosen as the initial global template set. Iteratively for all microstate template sets, the templates of each of these sets were spatially correlated with the templates of the initial global template set and assigned to one template based on the best spatial correlation values. A new initial global template set was then obtained by averaging all templates assigned to the same global template of the previous initial set. This sequence of spatial correlation/template assignment/template averaging was repeated with the new global template set, and a further initial global template set was obtained. The iteration of spatial correlation/template assignment/template averaging and definition of a new initial global template set was repeated until the best fit was found and a new iteration did not lead to any change in the definition of the global template set. With this procedure, four sets of global microstate templates were obtained (Maps in Figure 3). Lastly, a backfitting of the global templates was performed, as follows: for each subject and for each condition (EEG-Pre-Real, EEG-Post-Real, EEG-Pre-Sham, EEG-Post-Sham) the global microstate templates were backfitted to the EEG signals by calculating the spatial correlation between each global template and the scalp potential distributions at each GFP peak. With this procedure, each EEG time course was represented as a unique sequence of global microstates. For each of these sequences, the following metrics were calculated [14,28]:

- mean microstate duration (ms)—the average duration of each global microstate was calculated as the average time interval during which this microstate remained stable whenever it appeared [14];
- mean microstate occurrence (Hz)—the frequency with which each global microstate occurred, calculated as the average number of times per second that this microstate became dominant during the EEG time course; the microstate occurrence provides an indication of the tendency of the underlying neural generators to be activated and become dominant;
- mean microstate coverage (%)—for each global microstate, this metric was calculated as the fraction of the total recording time during which this microstate was dominant [14];
- global explained variance (GEV)—percent variance explained by each of the maps per experimental session (i.e., Real vs. Sham; at GFP peaks only).

#### 2.6.3. Power Spectral Density Analysis

The PSD was estimated for each EEG channel, for each subject, for each stimulation (REAL, for real stimulation and SHAM, for sham stimulation) and for each time (PRE, for before stimulation and POST, for after stimulation) by means of the Welch procedure (Hamming windowing of 8 s, resulting in a frequency resolution of 0.125 Hz, 50% overlap). For each EEG channel and for each condition, band powers were obtained by the sum of the power spectrum in each frequency band normalized by the number of frequency bins. The considered frequency bands were alpha (from 8 to 13 Hz), beta (from 15 to 25 Hz), theta (from 4.5 to 7.5 Hz) and delta (from 1 to 4 Hz).

#### 2.6.4. Statistical Analysis

Paired Wilcoxon sign-rank tests were carried out to assess significant differences of power spectrum activity for standard frequency bands (from delta to beta) across EEG channels for different conditions (Pre-Real vs. Post-Real; Pre-Sham vs. Post-Sham). Post hoc comparisons were corrected using the false discovery rate (FDR) method [29]. The differences of microstate metrics (microstate duration, occurrences per second, percentage of covered analysis time) among groups were evaluated by paired Wilcoxon sign-rank tests to assess the significant differences in each microstates’ metrics for different conditions (Real Pre vs. Real Post; Sham Pre vs. Sham Post). As the control analysis, paired Wilcoxon sign-rank tests were carried out to assess significant differences between baseline conditions (Pre-Real vs. Pre-Sham) and between stimulation conditions (Post-Real vs. Post-Sham) to also account for possible placebo effects. Paired Wilcoxon sign-rank tests were also employed to assess the significant differences of safety parameters (HR, systolic BP, diastolic BP) and the amplitude of electrical stimulation between real and sham tVNS. The significance level was set at *p* < 0.05. Results are reported as median and interquartile range [IQR], unless differently stated.

## 3. Results

### 3.1. Safety Measures and Tolerability

No major adverse events were registered during the experimental sessions. The cardiovascular physiological parameters stayed within the safety range throughout the entire experimental procedure for all eight subjects. More specifically, during the real tVNS the mean HR was 80.7 ± 16.8 bpm, the mean systolic BP was 122.4 ± 6.8 mmHg, and the mean diastolic BP was 79.5 ± 3.5 mmHg across all subjects. During the sham tVNS the mean HR was 75.4 ± 12.5 bpm, the mean systolic BP was 119.7 ± 6.4 mmHg, and the mean diastolic BP was 80.9 ± 2.7 mmHg across all subjects. No significant differences in safety measures were found between real and sham tVNS (*p* > 0.05 for all parameters).

The amplitude of electrical stimulation was kept constant during the entire experimental procedure after the initial adjustment for all patients. The mean stimulation amplitude was 6.8 ± 1.2 mA for real tVNS and 7.5 ± 2.4 mA for sham tVNS. No significant differences in stimulation amplitude were revealed between real and sham tVNS (*p* > 0.05). Adverse events were mild, and they were only reported during the initial phase of adjustment of the electrical stimulation (first 5 min). They included itching or burning sensations at the site of stimulation and ear pain in both sham and real tVNS, spontaneously resolving after the adjustment of the electrical stimulation’s amplitude.

### 3.2. Microstate Analysis

The optimal number of templates for each condition was four, according to the criteria for optimal number of templates. The global explained variance (GEV) was 73% for the Pre-Real for condition, 74% for the Post-Real condition, 76% for the Pre-Sham and 74% for the Post-Sham condition. No significant GEV differences were found among conditions. Studying the four templates across time and stimulation conditions, no differences were found between the microstates’ templates’ occurrences per second and the percentage of covered analysis time (*p* > 0.05 for both Real and Sham conditions). The analysis of the templates’ mean duration revealed significant differences only for the map A, which varied significantly after Real tVNS stimulation (69.1 (67.8–75.2) ms for Pre. vs. 74.6 (68.4–77.5) ms for Post., *p* = 0.03, effect size (r) = 0.58), while no differences were found after Sham tVNS stimulation (*p* > 0.05, Figure 3). The comparison of Post Real with Post Sham tVNS also revealed significant differences in mean Microstate A duration (74.6 (68.4–77.5) ms for Real and 64.1 (63.4–67.3) ms for Sham; *p* = 0.02).

### 3.3. Power Spectrum

**Figure 4:**
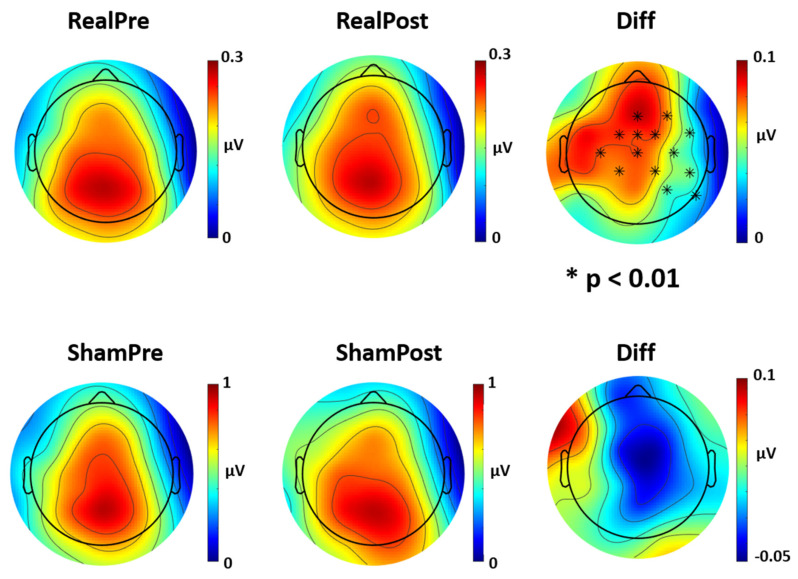
Topoplot of differences in delta power spectrum activity after Real and Sham tVNS stimulation. Delta power spectrum for Real and Sham condition, pre and post stimulation. Real tVNS induced an increase in delta activity across several channels (*p* < 0.01), while sham stimulation had no effect on delta activity. Diff = difference in Pre vs. Post stimulation.

The analysis of the four frequency power spectra (from delta to beta) across time and stimulation conditions found no differences between power spectrum values for theta, alpha and beta frequencies (*p* > 0.05 for both Real and Sham conditions). The analysis of the delta power spectrum revealed significant differences after Real tVNS stimulation for several EEG channels (FZ, FcZ, Cz, F4, FC2, FC6, C4, CP2, CP6, P4, P8, C3, FC1, CP1; *p* < 0.01 for Real Pre vs. Real Post, Figure 4), while no differences were found after Sham tVNS stimulation (*p* > 0.05, Figure 4). The comparison of stimulation conditions (Real Post vs. Sham Post) also revealed significant differences in delta activity in several channels (FcZ, FC1, FC2, Cz, C4, CP2; *p* < 0.05). No significant differences between baseline conditions were found (Real Pre vs. Sham Pre; *p* > 0.05).

## 4. Discussion

In this study, we aimed to characterize microstate metrics and quantitative EEG responses in relation to tVNS in a population of healthy subjects. Our results showed that tVNS seems to modulate EEG activity and microstate metrics, while sham stimulation had no effect. In particular, we observed that real tVNS induced (i) an increase in the metrics of microstate A mean duration and (ii) an increase in EEG power spectrum activity in the delta frequency band (1–4 Hz).

### 4.1. Microstate Metrics

The increment of microstate A mean duration suggests an increased stability of microstate A induced by real tVNS. There is no general agreement on the functional significance of microstate A and on its relationship with resting state networks [13,30,31,32]. On this issue, Britz and colleagues observed a correlation between functional magnetic resonance negative BOLD activations in bilateral superior and middle temporal gyri and microstate A, areas that are mainly implicated in phonological processing within the auditory networks [30]. However, recent studies suggested additional sources of microstate A in the frontal and parietal cortex [28], including the pre-supplementary motor cortex, medial superior frontal cortex and medial superior parietal cortex [33]. Taken together, these findings suggest the involvement of diffuse long-range global networks for microstate A, which may involve a close and mutual connection between “sensory” (auditory) networks and motor responses. Indeed, Gschwind and colleagues [34] investigated 53 patients with relapsing-remitting multiple sclerosis using high-density EEG, observing increases in the duration and appearance of microstate A and B, suggesting that multiple sclerosis may affects the “sensory” networks, rather than high-functional networks, as observed in schizophrenia [35]. Similarly, we may speculate that tVNS induces a disturbance across low-functional sensory-motor primary networks, which in turn may facilitate the spread of pathological networks (i.e., epileptic networks) through different symptomatic cortical areas [36]. Interestingly, the effect of tVNS on the primary sensory-motor cortex has already been investigated by Kraus and colleagues by means of functional magnetic resonance. They found a significant BOLD signal increase in the precentral gyrus of both sides induced by tVNS, suggesting a general activation of synaptic activity in the primary motor area induced by tVNS [8]. This is in line with our hypothesis of microstate A as a functional neurophysiological correlate of primary sensory-motor networks. These findings may eventually endorse the research into functional neurophysiological biomarkers of tVNS and VNS activation in specific and focused cortical areas (i.e., pre-central and post-central gyrus).

### 4.2. Power Spectrum Activity

Delta activity was significantly higher after real tVNS across several, widespread EEG channels, while showing no modification after sham stimulation. Delta waves (1–4 Hz) are the most prominent EEG feature of human non-rapid eye movement (NREM) sleep, which have their origin in the cortical layer. Several studies proposed them as sensors for weighing synaptic efficacy and possible effectors of sleep-dependent synaptic plasticity [37,38]. Furthermore, wakefulness delta power increase was correlated with TMS-induced LTP-like plasticity in healthy subjects [37] and in patients with chronic stroke [39], as well as with local sleep regulation, functionally linked to learning-related cortical plasticity [40]. Delta waves are almost absent in the physiological condition during wakefulness, but they tend to be largely expressed when a subcortical brain lesion occurs [41]. On the contrary, non-lesional delta waves are assumed to originate from a higher number of synchronous oscillating neurons or from a stronger activity of such neurons. Both of these hypotheses converge in the idea of focused information processing, which in turn may aim to induce local or network plasticity [37]. Delta waves are also tightly coupled with interictal high-frequency oscillations (HFOs) in patients with epilepsy [42]. Such slow-waves co-occurring with interictal HFOs might reflect the hyperpolarization of cortical neurons driven by thalamocortical networks, as suggested by Steriade and colleagues, who elegantly showed that the genesis of macroscopic delta EEG potentials depends on the proprieties of thalamocortical networks and brainstem–thalamic cholinergic modulation [43]. These evidences support the hypothesis of subcortical deep generators for delta oscillations in the human brain, which may also be influenced by the activity of brainstem nuclei. As such, the augmentation in delta activity observed after tVNS may be explained by the subcortical activation of brainstem structures linked to the vagus nerve. In particular, the nucleus of the tractus solitarius projects to the locus coeruleus and to the raphe nuclei, which provide widespread serotoninergic innervation to the neocortex.

Serotonergic neurotransmission is strongly related to the wake/sleep cycle [44]. The dorsal raphe serotoninergic activity is seen as an arousal system, being turned more or less off during sleep. Indeed, antidepressive selective serotonin re-uptake inhibitors (SSRIs), which increase the serotonin released at serotonergic terminals by inhibiting its reuptake, consistently produce a reduction of REM sleep and a synchronizing effect, with the reinforcement of slow-waves sleep reported in animals [44]. Moreover, the presence of GABAergic function in the dorsal raphe neurons has long been recognized [45]. This is in line with our previous work showing an activation of GABA-A inhibitory circuits following tVNS in healthy subjects, which was coupled with a decrease in cortical excitability evaluated with paired-pulse TMS [9,18]. Taken together, these findings suggest that the mechanisms behind tVNS and VNS are mainly mediated by complex interactions with subcortical inhibitory circuits and cortical excitability. Therefore, the increase in delta activity may possible represent an indirect consequence of such activation of inhibitory subcortical circuits.

### 4.3. Limitations and Future Directions

Our study has some limitations that should be stated. Data were collected in a quite small sample and during quite short resting state conditions; we maximized to investigate effects specific to tVNS by using effect size statistics and post-hoc multiple comparisons. It is also important to consider that, in order to provide the clinical implications of the present findings, future studies should investigate and validate the modulation of quantitative EEG parameters in patients with different neuropsychiatric conditions. Indeed, the inclusion of reliable EEG biomarkers in clinical tVNS studies could provide important information on the strength of its predictive value for clinical outcomes. As an example, in patients with epilepsy, invasive VNS was found to increase the P300 magnitude only in those who presented a significant reduction in seizure frequency [46]. Observing similar dynamic changes in EEG parameters induced by tVNS (increase in delta power and microstate A duration) may possibly reveal the same prognostic information for patients with epilepsy. Similarly, determining the EEG effects of tVNS in patients with depression over long periods of treatment may determine whether individuals with good responses (i.e., improved depressive symptoms) and those with a lack of response to tVNS and VNS therapy respond differently.

## 5. Conclusions

In conclusion, this study confirms that tVNS is a reliable and effective way to stimulate the vagus nerve, and the mechanisms of action of this activation can be successfully studied using scalp EEG quantitative metrics. Future studies are warranted to explore the clinical implications of these findings, and to focus the research of prognostic biomarkers of VNS and tVNS therapy for patients with drug-resistant epilepsy and other neuropsychiatric disorders.
